# Adolescent and young adult (AYA) oncology: A credentialed area of focused competence in Canada

**DOI:** 10.1002/cam4.5024

**Published:** 2022-07-13

**Authors:** Amirrtha Srikanthan, Jolanta Karpinski, Abha Gupta

**Affiliations:** ^1^ Division of Medical Oncology Department of Medicine University of Ottawa Ottawa Ontario Canada; ^2^ Division of Medical Oncology The Ottawa Hospital Cancer Centre Ottawa Ontario Canada; ^3^ Ottawa Hospital Research Institute Ottawa Ontario Canada; ^4^ Royal College of Physicians and Surgeons of Canada Ottawa Ontario Canada; ^5^ Department of Medicine University of Ottawa Ottawa Ontario Canada; ^6^ Princess Margaret Cancer Centre The Hospital for Sick Kids Toronto Ontario Canada

**Keywords:** adolescent and young adult, curriculum, education, oncology, training

## Abstract

**Background:**

Adolescents and young adults (AYA, ages 15–39 years) affected by cancer have unique treatment, survivorship, and palliation concerns. Current oncology training does not focus on the distinctive needs of this demographic. Amid this recognition, the Canadian National AYA Cancer Task Force and Canadian Partnership Against Cancer have advocated the need for clinicians with formalized AYA experience. To address this need and standardize training, a national task force developed criteria for structured academic programs in AYA Oncology in Canada.

**Methods:**

Workshops were organized to identify and establish the fundamentals of practice in AYA Oncology through consensus. These workshops followed the pre‐existing rigorous process established by the Royal College of Physicians and Surgeons of Canada (Royal College) for new program development. The process includes: (i) developing the tasks associated with the discipline's practice, (ii) identifying the evidence trainees must provide to demonstrate tasks can be performed independently (the competence portfolio), (iii) developing training requirements and summarizing the knowledge, skills and attitudes required to perform these tasks, and (iv) identifying specific experiences essential to acquiring skills and demonstrating competent performance.

**Results:**

AYA Oncology is a recognized an Area of Focused Competence (AFC) by the Royal College.

**Conclusion:**

The AFC designation in AYA Oncology provides a standardized curriculum, training experience and accreditation process to attract oncologists, promote expertise and advance AYA oncology care.

## Graphical abstract

The Formally Credentialed Area of Focused Competence Diploma designation through the Royal College of Physicians and Surgeons of Canada for Adolescent and Young Adult (AYA) Oncology provides a standardized curriculum, training experience and accreditation process to attract oncologists, promote expertise and advance AYA oncology care.

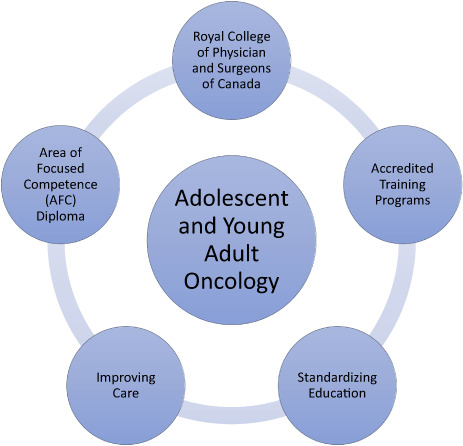

## 1 INTRODUCTION

Adolescents and young adults (AYA) – defined in North America as individuals between the ages of 15–39 years of age^1^ – are in a period of key development for milestones fundamental to a person's overall lifelong well‐being.^2^ These milestones include the development of values and personal identity, the formation of strong personal relationships, including romantic and sexual, establishing one's career and attaining financial independence.^3^, ^4^, ^5^, ^6^, ^7^ A cancer diagnosis disrupts this development, whether through facing early death, interruption of social life activities, returning to live with parents for care, fearing for the future due to treatment late effects or recurrence, and palliation.^3^, ^7^ Families of AYAs with cancer also experience distress, which may compromise their ability to support their AYA family member.^8^, ^9^

AYA oncology care requires a focus on ‘patient/family‐centred care’ – to give patients and their families the time and space to express all their needs and provide ongoing care throughout their cancer journey, whether into survivorship or end‐of‐life care.^8^, ^10^ A key part of providing this care is ensuring health care providers understand the developmental, fertility, and mental health impacts on this demographic, but also importantly, assist patients in addressing and coming to terms with the impact of an illness that appears early in one's life.^11^ In addition, treatment‐decision making is more challenging for AYA patients, as the decision making is more complex (due to the unique stage of the individual's life), and requires ongoing multifaceted reassessment and participation.^12^ Nearly one fourth of patients express regret about initial treatment decisions twelve months after such decisions are made.^13^

Given these distinctive needs, this demographic is not well served by traditional pediatric or adult models of care, leading to increases in AYA program development in many jurisdictions.^10^, ^14^, ^15^ The current training of oncologists does not focus on the distinctive needs of this demographic leading to a gap in care delivery, program development and advocacy.^10^, ^11^, ^15^, ^16^, ^17^, ^18^ For example, cancer patients may attain better survival outcomes on pediatric‐based protocols as opposed to adult‐institution protocols, as is the case for acute lymphoblastic leukemia.^19^ AYA patients with other cancers commonly seen in the pediatric population may also benefit from consultation with a pediatric oncologist. The importance of integrating pediatric oncology input for younger adults and enhancing communication between pediatric and adult institutions is critical. AYA oncologic care requires improving the quality of the cancer journey for AYA patients, which also requires a community of advocates to increase awareness and access to basic AYA needs, such as oncofertility and mental health support.^10^

Recognition of these needs led to an initiative from the consortium of children's cancer centers in Canada.^20^ In 2008, the executive approached the Canadian Partnership Against Cancer (CPAC), a federally funded organization tasked with implementing a pan‐Canadian strategy for cancer control.^20^ CPAC agreed that the AYA population was poorly served and understudied, and that organizing a national task force (NTF) to address these issues fell within its mandate.^21^ One of the solutions proposed by the Canadian AYA NTF was to improve training, recruitment and retention of trained AYA specialists.^20^, ^21^ The Canadian Partnership Against Cancer reinforced this in the Canadian Framework for the Care and Support of Adolescents and Young Adults with Cancer where a key supporting platform for enabling the strategic priorities listed is a “Workforce trained to address the needs of AYA with cancer”.^22^

Although AYA Oncology is a distinct area of practice within oncology, formal training opportunities are limited. Within Canada, one post‐residency fellowship opportunity exists.^23^ Within the US, fellowship opportunities have been developed in addition to online and in‐person personal training modules or courses that can be taken to enhance one's knowledge.^24^, ^25^, ^26^ The existing fellowship opportunities are neither accredited nor consistently tailored to the trainee's prior experiences and future goals. The quality of experiences of these programs are not externally validated like other oncologic specialties.^27^ In the context of health professional education, accreditation enhances health care outcomes by standardizing the quality of training programs, continually enhancing curriculum to align with evolving population needs and improving learning environments.^28^

These limited opportunities lead to a shortage in AYA dedicated specialists needed to advocate, design, and deliver tailored AYA oncologic care. Accreditation organizations, health care facilities, physician licensing bodies, and the public expect that physicians who practice will have formal training in their field.^29^ As such, the Royal College of Physicians and Surgeons of Canada (Royal College) has recognized an Area of Focused Competence (AFC) in Adolescent and Young Adult Oncology for enhanced training. This AFC provides standards for training, in addition to formal credentials and the designation of Diplomate to successful candidates.^29^ This paper serves to describe the process of developing formal accredited training opportunities in AYA Oncology.

## 2 METHODOLOGY FOR THE AFC DEVELOPMENT

### 2.1 Royal College of Physicians and Surgeons of Canada

The Royal College was established in 1929 by a Canadian Act of Parliament to oversee postgraduate medical education in Canada.^30^ The primary objective of the Royal College is to maintain the highest standards of specialist training and specialist care for Canadians. The Royal College is engaged in a broad spectrum of work ranging from standard setting for specialty education to the accreditation of post‐secondary specialty residency programs at universities. Continuing medical education requirements for specialists is overseen by the Royal College through its Maintenance of Certification program. The Royal College includes all medical and surgical disciplines except Family Medicine, which is governed separately by the College of Family Physicians of Canada (CFPC).^29^

It was recognized that for specific areas of clinical expertise, training experiences and confirmation of required skill sets was varied. There were often lack of nationally or internationally recognized standards. The Area of Focused Competence (AFC) designation was established by the Royal College to create a mechanism to standardize curriculum, training experiences and accreditation for trainees and programs to improve quality, maintain public safety and meet the needs of the public, institutions, and trainees.

### 2.2 The adolescent and young adult oncology application process

To be recognized as an AFC field of practice, Royal College criteria must be met. These criteria include demonstration that the field: (1) encompasses a distinct body of knowledge and a defined scope of practice, (2) is separate from and not negatively affecting existing and related disciplines, (3) serves a recognized health need that is not currently being satisfied by other disciplines, and (4) makes a positive contribution towards improved medical care and health outcomes.

Before an AFC can be recognized, at least one training site in Canada must be capable of developing a training program for the country, with the available resources. This criterion is to ensure there is adequate infrastructure to support the discipline. Additionally, at the time of AFC application, there must be a sufficient cohort of experts available to provide a high‐quality educational experience and an existing professional organization capable of advancing the field.^31^

The application for recognition of AYA Oncology as an AFC was first submitted in 2012 by The University of Toronto, Toronto, Ontario, Canada and University of Alberta, Edmonton, Alberta, Canada with endorsement by the Canadian Task Force on Adolescents and Young Adults with Cancer. AYA Oncology was recognized as an AFC in 2013 after the application underwent review by the Royal College Committee on Specialties, which includes a national consultation process.

### 2.3 Developmental process of the area of focused competence in AYA oncology

Once the AFC was recognized, a working group was created based on key stakeholders to evaluate the initial proposal co‐developed at the University of Alberta and University of Toronto. The goal of this evaluation was to ensure the standards of the Royal College were met and to ensure national applicability and adoption. The AFC Working Group for AYA Oncology initially met in 2013. This group consisted of the Chair, Vice‐Chair, and working group members from adult and pediatric oncology programs across Canada. The working group was supported by an AFC administrator and a clinician educator who is also the Associate Director of the Specialties Unit from the Royal College. A series of web conferences and in‐person meetings were held from 2013 to 2015. These conferences followed the rigorous process for competency‐based education curriculum development established by the Royal College.^32^, ^33^

The overall purpose of this process was to review, identify, establish, and agree upon the core elements of AYA Oncology. The process is well described by the Royal College.^29^ First, the major tasks required in the practice of the discipline are identified, in addition to the evidence a trainee must provide to demonstrate that they can perform these tasks independently (the Competency Portfolio). Next, the competency training requirements are developed, which summarize the knowledge, skills and attitudes required to perform these tasks. Lastly, the specific experiences required during training that are essential to acquire the required skills and demonstrate competent performance are described.

This process also included web conferences to finalize entry routes into the discpline, Competency Training Requirements, the Competency Portfolio and the Standards of Accreditation. These documents collectively define the discipline within the Royal College and were finalized in 2016.

### 2.4 Implementing the area of focused competence in AYA oncology

#### 2.4.1 Entry routes

Entry is possible from adult Medical Oncology, Radiation Oncology, Hematology or Pediatric Hematology/Oncology. The candidate must be eligible for Royal College certification in one of the four listed disciplines, or equivalent. Both Canadian and international candidates are eligible for training; the term “equivalent” extends eligibility to graduates of the four entry disciplines from all jurisdictions.

These entry routes were determined by the AYA AFC working group as the physicians who currently are delivering oncology care to those within the AYA demographic. Although there are mandatory competencies each trainee must demonstrate, the training experience will be tailored to the individual based on their prior training and experiences. For example, an adult trained oncologist will require additional experiences at pediatric institutions, a minimum of two month, compared to a trainee from pediatric oncology. Similarly, a pediatric trained oncologist will require additional experiences, a minimum of two months, in adult cancer programs. The AYA Oncology AFC Program Committee at the university hosting the program provides oversight for the fellowship program, and ensures that each candidate is provided the experiences and opportunities to supplement and enhance their knowledge and successfully complete the fellowship.

#### 2.4.2 The competency portfolio and standards of assessment

The Major Tasks of the AYA Oncology scope of practice were determined and are:

1. Evaluation and management of AYA patients throughout all stages of the cancer journey
2. Clinical leadership of the interprofessional team caring for AYA patients with cancer
3. Advancement of the discipline of AYA Oncology

The Competency Portfolio describes each of the Major Tasks of the discipline and outlines the corresponding milestones the trainee should perform independently to become a Diplomate in the discipline. The first major task follows the AYA patient from diagnosis, on treatment, at conclusion of active therapy and into survivorship or though end‐of‐life care. The second major task highlights the role of the AYA oncologist as a bridge between the pediatric and adult health care systems, and as a leader and advocate for the AYA oncology population. The third major task emphasizes the need to advance the discipline through enrollment in clinical trials, through scholarly advancements in diagnosis, treatment and health care delivery, and through education of other health care professionals.

The standards of assessment outlines the information and documentation required by the trainee as evidence to support that the individual has successfully acquired the abilities associated with each milestone.

For universities that create accredited AYA Oncology fellowship programs, trainees would undergo rotations in various fields relevant to AYA, including: oncofertility, survivorship, psychosocial oncology, palliative care, concurrent theme‐based blocks (such as cancer genetics) and disease specific rotations (tailored to the fellows interests and career goals, such as leukemia, lymphoma, sarcoma, testicular cancer etc.). Rotations include both experiences at both pediatric and adult institutions, therefore the AYA Oncology AFC Program Committee and teaching staff requires staff and personnel at both institutions to establish the training opportunity and provide oversight to the program.

#### 2.4.3 Competency training requirements

To successfully perform the Major Tasks and milestones, the trainee must acquire a working knowledge of both the clinical and theoretical basis of the discipline, including its foundations in science and research, consistent with other approved disciplines.^29^ The list of required competencies was developed according to the CanMEDS competency framework (https://www.royalcollege.ca/rcsite/documents/ibd/oncology‐aya‐afc‐ctr‐e).^34^, ^35^ This framework includes the roles of Medical Expert, Communicator, Collaborator, Leader, Health Advocate, Scholar and Professional.^34^, ^35^

The required knowledge and skills emphasize key elements of practice for a physician in the AYA Oncology AFC discipline, such as: application of the sciences relevant to AYA Oncology (cancer genetics, hereditary cancer syndromes); risk‐mitigation strategies to preserve function and provide surveillance for short‐ and long‐term side effects (such as cardiac, endocrine, neurocognitive, fertility health and secondary malignancy risk); holistic quality of life assessments including factors such as education, interpersonal skills, and risky behaviors; and palliative care implications. In addition, non‐clinical roles were identified, including participation in academic activities (such as program development, research and teaching), and development and/or oversight of collaborative initiatives to improve AYA Oncologic care.

Recognizing that oncofertility is a very large part of AYA care, enhancing medical knowledge in oncofertility is a competency training requirement. As such a required training experience includes the participation in oncofertility and sexual health consultations to understand the breadth and variety of available treatments. These consultations and clinical experiences will be undertaken at pediatric and adult cancer institutions. Each AFC program committee also includes reproductive specialists (such as gynecologists and urologists) who can offer additional experiences at fertility clinics and processing facilities should the trainee wish to pursue this.

Successful AYA Oncology Diplomates will have demonstrated through their portfolio, with confirmation by training programs, that the candidate has acquired the knowledge and skills necessary for independent practice. The AYA oncology competencies can be found at https://www.royalcollege.ca/rcsite/ibd‐search‐e. Required and Recommended Training Experiences are listed in Table 1.^36^

**TABLE 1:** Required and recommended training experiences

REQUIRED TRAINING EXPERIENCES
Perform clinical assessments and manage the ongoing care of AYA patients with cancer in an ambulatory clinic setting at all stages of the cancer journey Act as an AYA Oncology consultant for ambulatory and hospitalized patients Participate in oncofertility and sexual health consultations to understand the breadth and variety of available treatments Participate in palliative care consultations in the pediatric or AYA age group, either in the inpatient or ambulatory setting Participate in psychosocial oncology consultations for AYA patients Perform clinical assessments of AYA patients in a survivorship/late effects clinic Teach AYA Oncology topics for a variety of audiences Participate in multidisciplinary tumor board rounds at both adult and pediatric centres Participate on a committee or action group relevant to AYA Oncology at the local institution, or at a provincial or national level Participate in a scholarly project
RECOMMENDED TRAINING EXPERIENCES
Attend a conference relevant to AYA Oncology Complete a scholarly project and submit an abstract for a relevant meeting Participate in the clinical care of AYA patients with cancer outside the scope of the AFC trainee's entry discipline (Pediatric Hematology/Oncology, Radiation Oncology, Medical Oncology, Hematology) Participate in the process of transitioning patients to an alternate level of care (e.g., to aftercare/survivorship from active care, or to adult setting from pediatric)
Copyright © 2016 The Royal College of Physicians and Surgeons of Canada. Referenced and produced with permission.

#### 2.4.4 Standards of accreditation

Standards of accreditation are the requirements a university must initiate and maintain to be approved for a training program in the discipline. Standards include describing the resources needed for training, including access to the appropriate patient population. It also includes organizational standards such as the infrastructure to support program administration, teaching, and assessment. Training programs must meet accreditation standards for their trainees to achieve Royal College recognition as a Diplomate.^29^

#### 2.4.5 Funding

The training program in AYA Oncology can be funded from various sources, including: (i) provincial budgets intended for postdoctoral training programs at medical faculties, with institutional support dependent on the jurisdiction, (ii) national professional organizations and (iii) pharmaceutical sponsorship. International physicians may be sponsored for their training by their home country or through a training grant. At present, dedicated funding does not exist at a national level for these fellowships; however, individual institutions hosting the programs may have internal funds to support prospective trainees.

#### 2.4.6 Practice eligibility route to certification

The practice eligibility route (PER‐AFC) allows physicians who are currently working, and have gained expertise in the discipline through their own training and practice to apply for the AFC designation. These individuals may have completed their training prior to the establishment of the AFC or through informal fellowship opportunities. These applicants are established and competent physicians in the field and are certified based on demonstration of this competence. Specifically, an application is submitted where the candidate demonstrates that they have met the requirements of the AFC. As part of the application, a multisource feedback questionnaire is sent to referees attesting to the candidate competence in the major tasks of AYA Oncology and their current scope of practice as a AYA Oncologist. Applicants through this stream must have been in practice for a minimum of 5 years. Successful candidates receive the Diplomate and AFC designation.

#### 2.4.7 Implementation

The Competency Training Requirements, the Competency Portfolio and the Standards of Accreditation documents went live in 2016, enabling the creation of AYA Oncology programs at Canadian post‐secondary medical education institutions. The University of Toronto submitted an application for an AYA Oncology program, which was approved, and accredited in 2020. The PER‐AFC route documents were developed in 2021 and was opened to applications January 4, 2022.

## 3 DISCUSSION

Adolescents and Young Adults (AYA) affected by cancer face a range of distinct issues that are currently not well addressed by pediatric or adult institutions.^10^, ^21^ These include, but are not limited to: sexual health, return to school/vocation challenges, re‐integration into social environments appropriate for maturational stage, and increased medical concerns such as fertility, mental health, late side effects from cancer therapy and secondary malignancies.^10^, ^22^ In addition, challenges remain for these patients regarding treatment decision‐making, challenges that can be greatly ameliorated by increased trust and mutual understanding in the patient‐oncologist relationship.^13^ Ensuring health care providers exist that are attune and ready to address these unique needs can improve AYA oncologic care.

While programs exist for children and older adults facing cancer, individuals between 15 and 39 years of age are not well supported during active treatment, survivorship or palliation. Key national and international organizations recognize the importance of clinicians with AYA training to ensure improved care delivery and outcomes for this demographic.^10^, ^20^, ^22^ In addition to increased training opportunities for clinicians, improved care for this population requires dedicated resource allocation for the creation of dedicated programs, including the physical infrastructure for such programs.^37^ Funding from hospitals and government agencies are essential for this care to be successfully delivered. Specialized training of AYA oncologists allows for the creation of health care providers who can continue to advocate for these patients and the required changes.

The current shortage of AYA clinicians, and ability to identify existing AYA specialists are two of the limiting factors for ensuring an optimal cancer experience.^22^ The creation of accredited structured training programs in AYA Oncology provides trainees with the current evidence‐based knowledge and competencies critical to their practice.

The AYA Oncology AFC has developed standards for the recognition of training in this specialized and growing discipline. Through providing standardized curriculum, training experiences and accreditation, graduates can demonstrate that formal training in the discipline has been obtained and expertise achieved. In addition, these physicians will become future leaders in AYA Oncology clinical care, research, and education in Canada and internationally, allowing for improved advocacy and development of AYA oncologic care.

## AUTHOR CONTRIBUTIONS

A.S. formulated the design of the article and wrote the first draft of the article. A.S., J.K., and A.G. critically reviewed subsequent versions and approved the final version.

## FUNDING INFORMATION

There are no funding sources to declare for this work.

## CONFLICTS OF INTEREST

The authors declare no competing financial interests or conflicts of interest.

## ETHICS APPROVAL

Ethics approval was not required for this work.

## PATIENT CONSENT STATEMENT

Patient consent was not required for this work.

## Data Availability

As a study was not conducted, data are not available for sharing. Details and dates presented in this manuscript are publicly available through websites, or reaching out to the Royal College of Physicians and Surgeons of Canada.
